# Immunomodulatory Properties and Molecular Effects in Inflammatory Diseases of Low-Dose X-Irradiation

**DOI:** 10.3389/fonc.2012.00120

**Published:** 2012-09-25

**Authors:** Franz Rödel, Benjamin Frey, Katrin Manda, Guido Hildebrandt, Stephanie Hehlgans, Ludwig Keilholz, M. Heinrich Seegenschmiedt, Udo S. Gaipl, Claus Rödel

**Affiliations:** ^1^Department of Radiotherapy and Oncology, University Hospital of Frankfurt, Johann Wolfgang-Goethe UniversitätFrankfurt am Main, Germany; ^2^Department of Radiation Oncology, University Hospital Erlangen, Friedrich-Alexander Universität Erlangen-NürnbergErlangen, Germany; ^3^Department of Radiotherapy and Radiation Oncology, University of RostockRostock, Germany; ^4^Department of Radiotherapy, Clinical Center BayreuthBayreuth, Germany; ^5^Strahlenzentrum Hamburg Medizinisches VersorgungszentrumHamburg, Germany

**Keywords:** discontinuous dose dependency, inflammation, immune modulation, low-dose radiation therapy

## Abstract

Inflammatory diseases are the result of complex and pathologically unbalanced multicellular interactions. For decades, low-dose X-irradiation therapy (LD-RT) has been clinically documented to exert an anti-inflammatory effect on benign diseases and chronic degenerative disorders. By contrast, experimental studies to confirm the effectiveness and to reveal underlying cellular and molecular mechanisms are still at their early stages. During the last decade, however, the modulation of a multitude of immunological processes by LD-RT has been explored *in vitro* and *in vivo*. These include leukocyte/endothelial cell adhesion, adhesion molecule and cytokine/chemokine expression, apoptosis induction, and mononuclear/polymorphonuclear cell metabolism and activity. Interestingly, these mechanisms display comparable dose dependences and dose-effect relationships with a maximum effect in the range between 0.3 and 0.7 Gy, already empirically identified to be most effective in the clinical routine. This review summarizes data and models exploring the mechanisms underlying the immunomodulatory properties of LD-RT that may serve as a prerequisite for further systematic analyses to optimize low-dose irradiation procedures in future clinical practice.

## Introduction

The relationship between ionizing radiation and an inflammatory response displays a dichotomous character and greatly depends on the radiation dose/quality and immune cell types investigated. When compared to a high dose exposure with pronounced inflammatory promoting effects (Williams et al., 2003), low-dose irradiation (single doses ≤1.0 Gy) reveals anti-inflammatory properties (Seegenschmiedt et al., 2008; Rödel et al., 2012). This implicates the involvement of complex mechanisms differentially operating at different dose levels (Marples et al., 2004). Although low-dose radiation therapy (LD-RT) for the treatment of inflammatory and degenerative diseases (Seegenschmiedt et al., 2008) is successful in clinical use since several decades, underlying immunological and molecular mechanisms are far from being fully explored, in part because of their unusual discontinuous dose dependency and non (DNA)-targeted properties. The present review focuses on immunomodulatory properties of LD-RT to document the anti-inflammatory efficacy with special emphasis on preclinical *in vivo* models.

## Clinical Application of Low-Dose Radiation Therapy

The first known description of the clinical implementation of LD-RT to treat patients with non-cancerous diseases was as early as 1898 when Sokoloff and Stenbek reported on pain relief in patients with juvenile arthritis (Schmid-Monnard, 1898; Stenbek, 1898). More than 100 years later, a pattern of care study performed in Germany was published with 37,410 patients treated for inflammatory diseases proving LD-RT to be an accepted conservative treatment option at least in this country (Seegenschmiedt et al., 2004). Concepts and doses in clinical practice have been established empirically in the early twentieth century (von Pannewitz, 1933) recommending local treatment with single doses of 0.3–1.0 Gy in 4–5 fractions for acute and 1–3 fractions for chronic inflammatory disorders adding to a total doses of 3–5 Gy (acute) and 12 Gy (chronic), respectively (Seegenschmiedt et al., 2008). Typical clinical indication comprise degenerative disorders like rotator cuff syndrome (impingement of the shoulder joint), tennis/golfer’s elbow (Epicondylopathia humeri), painful heel spur (plantar fasciitis), exacerbated refractory, and painful osteoarthritis, or hyper-proliferative syndromes like Dupytren’s disease or the prevention of heterotopic ossification (Seegenschmiedt et al., 2004, 2008). Concerning the most clinical relevant endpoints pain relief, response, and analgetic effects, LD-RT is reported to result in a 33–100%, a 47–100%, and a 12–89% efficacy, respectively (Kutzner et al., 2003; Micke and Seegenschmiedt, 2004; Niewald et al., 2007; Adamietz et al., 2010; Betz et al., 2010; Heyd et al., 2010). Moreover, due to the low-doses used in actual clinical practice, radiogenic acute or chronic side effects were not observed in the treatment of inflammatory diseases (Seegenschmiedt et al., 2008). By contrast, LD-RT is still considered unfashionable in some (Anglo-American) countries due to elder reports on harmful side effects and increased mortality from leukemia and anemia (Cannon et al., 1959; Court-Braun Wm, 1965). Nevertheless, non-steroidal or steroidal drugs used as a pharmaceutical alternative also display numerous side effects and a considerable number of patients does not respond to the treatment (Rainsford, 2007). Consequently, after improving radiation protection, LD-RT is practised on an increasing number of patients as an effective and safe treatment routine. To underscore this, a more recent pattern of care study (Mücke et al., 2010) reported on 4.500 patients with osteoarthritis of the knee receiving LD-RT demonstrating an increased acceptance (95% referral) of this treatment alternative in Germany.

## Principles of Inflammation

Inflammation comprises a complex and basic immunological response to harmful stimuli, such as pathogens, damaged cells, or irritants to remove the stimuli and to initiate a healing process. Moreover, inflammation is a stereotyped response, and therefore is considered to be a mechanism of innate immunity, in contrast to a pathogen specific adaptive immunity (Murphy, 2011). A cascade of biochemical events propagates and matures the inflammatory response, involving the local vascular system. A pivotal molecular mechanism in the regulation of the inflammatory response is the secretion of regulatory cytokines. Whereas interleukin-1 (IL-1) or tumor necrosis factor-α (TNF-α) activate cellular components in a pro-inflammatory manner, anti-inflammatory peptides like the isoforms of transforming growth factor β (TGF-β_1–3_) or IL-10 down regulate and thus limit the inflammatory cascade (Mosmann, 1994).

An early event in the inflammatory cascade is the recruitment of leukocytes from peripheral blood by activation of local endothelial cells (ECs) with pro-inflammatory mediators mainly produced by macrophages and dendritic cells (DCs) at the site of the damaged tissue (Speyer and Ward, 2011). The subsequent effector phase of inflammation is characterized by the accumulation of monocytes and their differentiation into DCs or inflammatory macrophages (Adams, 1989). These cells support the local inflammatory process by a plethora of functions like phagocytosis, cytotoxicity, antigen presentation, secretion of cytokines, and the production of nitric oxide (NO), or reactive oxygen intermediates (Ding et al., 1988). Inflammation can be further classified as either acute or chronic (prolonged), characterized by a progressive shift in the type of cells present at the site of inflammation and by simultaneous destruction of the tissue from the inflammatory process.

## Modulatory Properties on Endothelial Cells of Low-Dose Irradiation

As reported before, EC play a crucial role in the regulation of the local inflammatory process both by their ability to recruit leukocytes from peripheral blood and to express a variety of cytokines/chemokines and growth factors (Speyer and Ward, 2011). As a consequence, the effect of low-dose irradiation on the adhesion process was analyzed in *in vitro* assays using human (EA.Hy926) or murine (mlEND1) EC and peripheral blood mononuclear cells (PBMC). LD-RT prior to the stimulation by TNF-α resulted in a hampered adhesion of PBMC to 43–50% of the control level at 4 and 24 h but elevated values at 12 h after irradiation with a single dose of 0.3–0.6 Gy (Kern et al., 2000a; Hildebrandt et al., 2002; Rödel et al., 2002). This characteristic coincides with a biphasic kinetic and elevated expression of the anti-inflammatory cytokine TGF-β1 both on the levels of mRNA and protein in the same dose range. Moreover, abrogation of TGF-β1 by neutralizing antibodies restored adhesion of PBMC to irradiated EC (Rödel et al., 2004b), indicating the cytokine to be a key player in the modulation of adhesion following low-dose exposure. A hampered adhesion is further supported by a lowered expression of the adhesion molecule E-selectin on stimulated EC with a local minimum following a 0.3–0.5 Gy exposure. This indicates that the modulation of E-selectin may further contribute to the anti-inflammatory properties of LD-RT (Hildebrandt et al., 2002; Rödel et al., 2002).

## Modulatory Properties on Leukocytes of Low-Dose Irradiation

The major cellular elements of the immune system comprise different lineages of lymphocytes (B and T cells) as members of an antigen-specific effector response, as well as polymorphonuclear (PMN) and mononuclear leukocytes (PBMC) as components of the innate immune system (Kobayashi et al., 2005). As outlined below, these immune cells are modulated by distinct low-doses of X-ray.

Apoptosis is a physiological endogenous cellular suicide program mediated by a variety of endogenous and exogenous stimuli including ionizing irradiation (Hengartner, 2000). Beside its central role in cellular homeostasis, apoptosis significantly impacts on immune regulation and radiation response. In line with that, cells undergoing apoptosis contribute to the modulation of activated mononuclear cell activity in a paradox manner by reducing the secretion of pro-inflammatory cytokines like TNF-α or IL-1. In addition, secretion of the anti-inflammatory peptide IL-10 is increased indicating an immune-suppressive potential of apoptotic cells (Voll et al., 1997). In 1999, Kern et al. (1999) were the first to report on a dose-dependent discontinuous increase of apoptosis in PBMC with a plateau or peak between a 0.3 and 0.7 Gy exposure. Additionally, the coincidence of a reduced PBMC/EC adhesion (as reported before) and induction of apoptotic cell death in PBMC prompted the group to investigate a putative link to the expression of adhesion molecules on the surface of the PBMC. They described a time-dependent proteolytic shedding of L-selectin that was associated with their early apoptotic phenotype (Kern et al., 2000b). More recently, a comparable performance of apoptosis induction was reported for PMN irradiated 2 h before stimulation with phorbol myristate acetate (PMA). Applying subG1 DNA content analyses, a discontinuous appearance of cell death was observed, showing a relative maximum at 0.3 Gy and a minimum at 0.5 Gy, respectively (Gaipl et al., 2009). Notably, the discontinuous course of apoptosis parallels a diminished protein level of mitogen activated protein (MAP) kinases and protein kinase B (or AKT), reported to be involved in the regulation of proliferation, transcription, and apoptosis (Yang et al., 2004).

Neutrophilic PMN accumulation has been implicated in the pathology of acute and chronic inflammatory diseases, such as rheumatoid arthritis (Witko-Sarsat et al., 2000) in part by the secretion of chemotactic cytokines with the potential to amplify leukocyte infiltration (Scapini et al., 2000). Thus, the impact of LD-RT on chemokine secretion in PMN was analyzed. In comparison to CXCL8 and CCL18, CCL20 chemokine was shown to be exclusively induced in a TNF-α dependent manner by a cell–cell contact between PMN and EA.hy926 EC. Furthermore, irradiation with doses between 0.5 and 1.0 Gy resulted in a discontinuous reduction of CCL20 secretion that parallels a hampered PMN adhesion to EC with a pronounced effect at a 0.7 Gy exposure (Rödel et al., 2008).

Very recently, Bauer et al. (2011) reported that monocytes are severely impaired in base and DNA double-strand break repair that renders them highly vulnerable to ROS and irradiation induced cell death by apoptosis. Thus, it is tempting to assume that a selective killing of monocytes at doses below 1 Gy may cause a depletion of macrophages and DCs that may further contribute to the anti-inflammatory effects of LD-RT.

## Modulatory Properties on Macrophages and DCs of Low-Dose Irradiation

Monocytes, unlike PMN, differentiate into tissue resident DCs or macrophages (Adams, 1989). Due to their central role in the initiation and the resolution of inflammatory processes, these cells are considered as key players in the regulation of inflammation (Valledor et al., 2010). Macrophages for example support a local inflammatory process by a variety of functions including phagocytosis, antigen presentation, secretion of cytokines, and the expression of enzymes like inducible nitric oxide synthase (iNOS; Fujiwara and Kobayashi, 2005). The latter enzyme processes the synthesis of nitric oxide (NO) that in turn increases vascular permeability and is involved in inflammatory pain (Holthusen, 1997; Abramson et al., 2001). In that context, low-dose radiation (≤1.0 Gy), if applied before stimulation with lipopolysaccharide (LPS) and interferon-γ (IFN-γ) of murine RAW 264.7 cells (a mouse leukemic monocyte macrophage line) decreases iNOS protein and NO production without affecting iNOS mRNA expression (Hildebrandt et al., 1998). This may indicate a translational or post-translational regulation of the enzyme that is linked, at least in part, to the analgetic properties of LD-RT.

Tsukimoto et al. further examined signal transduction pathways in RAW264.7 macrophage cells following γ-irradiation (137Cs source) with doses of 0.5–1.0 Gy. Dephosphorylation of both extracellular-signal-regulated kinases 1/2 (ERK1/2) and p38 mitogen activated protein kinase (MAPK) was observed at 15 min after irradiation which was concomitant with a significant increase in the expression of the MAPK phosphatase-1 (MKP-1; Tsukimoto et al., 2009). Since activated p38 MAPK mediates pro-inflammatory cytokine expression, they further assayed the effect of low-dose radiation on TNF-α, showing that production of the cytokine induced by LPS was significantly suppressed in 0.5 Gy irradiated macrophages.

A further essential inflammatory cytokine is the IL-1 family member IL-1β, which shows numerous activities in the inflammatory process (Dinarello, 2011). Using RAW264.7 macrophages, a non-linearity in IL-1β production was observed after high-linear energy transfer (LET) carbon ion radiation (Conrad et al., 2009). Notably, as compared to other inflammatory cytokines, expression of IL-1β is tightly regulated by a three step process and requires two distinct stimuli (Tschopp et al., 2003). In more recent studies using human THP-1 derived macrophages which were stimulated by LPS and MSU (mono sodium urate crystals) a significantly decreased IL-1β secretion at doses of 0.5 and 0.7 Gy was confirmed by Lödermann et al. (2012). In their experiments, the IL-1β machinery (also called the NALP3 inflammasome) was not affected by the doses used, but a hampered secretion of the IL-1β correlates with a reduction in nuclear translocation of the transcription factor nuclear factor κB (NF-κB) subunit RelA (p65) in line with a decreased protein amount of upstream (p38 MAPK) and downstream molecules (AKT). Thus it is tempting to conclude, that the discontinuous regulation of IL-1β following LD-RT may occur in a NF-κB dependent manner.

Furthermore, activated macrophages are a major source of reactive oxygen species (ROS) when they mount an oxidative burst to destroy pathogens. Accordingly, the impact of low-dose irradiation on oxidative burst activity and superoxide production was investigated in RAW 264.7 macrophages after stimulation with TNF-α/IFNγ, PMA, or the yeast product zymosan. Low X-ray doses between 0.3 and 0.6 Gy significantly reduced the oxidative burst in these activated macrophages, whereas higher doses had little effect. This indicates that a diminished release of ROS may contribute to the local therapeutic effect of LD-RT (Schaue et al., 2002).

Finally, Jahns et al. recently analyzed the effect of LD-RT on the maturation, cytokine release, and T-Lymphocyte activation of human DCs. They indicated that irradiation of DC-precursors *in vitro* does not influence surface marker (CD80, CD83, CD86) expression or cytokine profile of immature DCs nor of mature DCs stimulated by LPS, neither did it influence the capacity of the DCs to stimulate T-cell proliferation (Jahns et al., 2011).

## Modulation of Transcription Factor Activity by Low-Dose Irradiation

As there is enormous evidence for the involvement of cellular transcription factors including TP53, activating protein 1 (AP-1), and NF-κB in both, cellular radiation response and inflammation (Habraken and Piette, 2006), they may also represent a crucial link between low-dose radiation and their immune modulatory properties. The family of NF-κB transcription factors comprises a heterogeneous group of homo- or heterodimeric members of the Rel family including p50, p52, p65/RelA, c-Rel, and RelB (Baeuerle and Baltimore, 1996; Oeckinghaus et al., 2011). NF-κB is located in the cytoplasm in an inactive form by binding to inhibitor molecules of the IκB family (IκBα, IκBβ, IκBγ/NEMO, IκBε, p100, and p105; Huxford and Ghosh, 2009). Upon dissociation of the inhibitors, NF-κB dimers translocate into the nucleus and bind specific sequence elements in the enhancer/promoter regions of a variety of effector genes. These include factors implicated in DNA damage repair, the execution, or inhibition of cell death by apoptosis (Oeckinghaus et al., 2011) and cytokines (e.g., IL-1, TNF-α), adhesion molecules (e.g., E-selectin), and enzymes (e.g., iNOS) essential in the regulation of the immune system (Kracht and Saklatvala, 2002). In the field of low-dose irradiation, Prasad et al. (1994) were the first to report on an activation of NF-κB in a discontinuous manner with peak activities at 8 and 36 h after irradiation analyzed in 244B lymphoblastoid and B16 melanoma cells. In accordance to these findings, a comparable time dependence of DNA binding and transcriptional activity with a first peak at 4 h and a second peak at 24–30 h was confirmed in stimulated human EA.Hy926 EC (Rödel et al., 2004a). Based on these initial observations, factors engaged in the pathway(s) of NF-κB activation in stimulated EA.Hy926 EC were investigated. Among these regulatory proteins, X chromosome-linked inhibitor of apoptosis protein (XIAP) that enhances RelA/p65 nuclear translocation and promotes the degradation of IkB (Hofer-Warbinek et al., 2000; Jin et al., 2009) was investigated in more detail. Following irradiation, a discontinuous profile of XIAP-expression was observed with a relative maximum at 0.5 and 3.0 Gy which parallels a discontinuity in NF-κB induction. Furthermore, RNA-interference (siRNA) derived knockdown of XIAP resulted in a hampered NF-κB transcriptional activity, indicating a regulatory interrelationship between these factors (Rödel et al., 2010). These findings are in agreement with the observation that XIAP interacts with MAP Kinase Kinase 2 (MEKK2). The latter has previously been shown to be associated with a second wave NF-κB activation and the propagation of inflammatory processes (Winsauer et al., 2008). Moreover, a functional consequence of XIAP-expression and altered NF-κB activity on the adhesion process was obvious since XIAP attenuation showed an abrogation of the reduced PBMC binding normally observed following a 0.5 Gy exposure. This effect is, at least in part, driven by a reduced secretion of the cytokine TGF-β_1_, a key player in the anti-inflammatory effects of low-dose irradiation (Rödel et al., 2007).

Members of the c-Fos and c-Jun protein family that collectively form the homo- or heterodimeric AP-1 complex (Criswell et al., 2003), are considered to be involved in the transcription of a variety of immune effector molecules, including TGF-β_1_. By applying electrophoretic mobility shift assays (EMSA) and luciferase based transcriptional activity assays, a biphasic induction of AP-1 was detected in EA.Hy 926 EC (Rödel et al., 2009) that may further contribute to the immunomodulatory properties of LD-RT.

## Mechanisms Underlying a Discontinuous Characteristic of Low-Dose Irradiation Effects

The classical paradigm of radiation biology is based on the concept, that deposition of energy to the nucleus and the resulting DNA damage is responsible for the biological consequences of radiation exposure. By contrast, based on recent findings, there is growing evidence for non-(DNA) targeted effects that challenged this classical concept. Among these findings bystander or distant out of field (abscopal) mechanisms, as well as adaptive responses have been reported (Mothersill and Seymour, 2006; Hildebrandt, 2010). Notably, these novel concepts also take into consideration a complex intercellular communication and describe radiation responses on a tissue level (Barcellos-Hoff, 2005). The molecular mechanisms responsible for the discontinuous dose response characteristics, a common hallmark of these non-targeted effects, remain elusive at present and most likely originate from an overlap of several processes that may be initiated at various thresholds and operate in a staggered manner. There may also be parallels to the phenomenon of low-dose hyper-radiosensitivity and induced radioresistance, which have been reported for cellular survival at doses below 0.3 Gy and in the dose range of 0.3–0.6 Gy (Joiner et al., 2001; Marples and Collis, 2008). The current hypothesis on the regulation of this behavior is that the HRS region (<0.3 Gy) reflects an area of increased induction of apoptosis in cells that failed to undergo an ataxia telangiectasia-mutated (ATM)-dependent G2-phase cell cycle arrest. By contrast, a transition to induced radioresistance originates from a shift toward a G2-checkpoint induction, giving time for repair of DNA damage, and to increase cell survival. Corresponding to this, DNA double-strand breaks induced after very low-dose irradiation do not seem to be repaired, and cells containing residual damage were removed by a TP53-dependent apoptosis (Rothkamm and Löbrich, 2003). Interestingly, two seminal molecular studies (Xu et al., 2002; Bakkenist and Kastan, 2003) have shown discontinuous responses over the 0.1–1.0 Gy dose range, the most important being the activation and (auto)phosphorylation of the DNA damage sensor ATM. Once activated, ATM is implicated in several signaling cascades and is essential for the regulation of a cell cycle arrest, DNA damage repair, and acts as an inducer of the ATM–IκK–NF-κB signaling pathway (Hacker and Karin, 2006).

Based on these findings, it is reasonable to assume that beside DNA (repair)-mediated mechanisms, a non-linear dose-effect relationship may be associated with a differential protein expression. In line with that, Pluder and colleagues recently reported that exposure to Co^60^ γ-rays of EA.hy926 EC resulted in rapid change in the cytoplasmic proteome. The group identified 15 significantly differentially expressed proteins of which 10 were up- and 5 down-regulated. Pathways influenced by the factors include the RhoA pathway, fatty acid metabolism, and cellular stress response (Pluder et al., 2011).

## Anti-Inflammatory Properties in Preclinical Models of LD-RT

Beside an increasing knowledge concerning underlying mechanisms, *in vivo* models of experimentally induced arthritis (Gilroy et al., 1998) have been established to investigate a clinical anti-inflammatory efficacy of LD-RT. In 1933 von Pannewitz (1933) reported on a first series of animal studies, when rabbits with electro-coagulation of the knee joint cartilage or by mechanical bone destruction were treated with 1.0 Gy single dose irradiation. This treatment does not show a benefit on the degenerative changes, however, an improvement of the symptoms joint swelling (an indicator of reduced inflammation) and pain was observed. In subsequent years a variety of inducible models have been established to more closely mimic the situation in joints of patients suffering from rheumatoid arthritis (Smolen and Steiner, 2003). Experimental induction of arthritis in rodents treated either with zymosan (a yeast product) or inactivated mycobacterium tuberculosis (mtb) results in a fast (five days from injection) joint swelling associated with cartilage destruction and bone loss (Asquith et al., 2009). Using an intra-articular injection of papain, inactivated mtb or zymosan, Budras et al., Trott et al., and Fischer et al. induced an acute arthritis in rabbit knees. In their experiments five weekly fractions of 1.5 or 1.0 Gy significantly diminished the inflammatory proliferation of the synovial cover cells, the synthesis of synovial fluid, and thus swelling of the joint (Budras et al., 1986; Trott et al., 1995; Fischer et al., 1998). Although partially not reaching a level of significance, morphometric data further revealed a decreased number of the synovial cell layers and thus thickness of the synovial membrane and a lowered distance between capillaries and synovial membranes following irradiation.

The effects of LD-RT on morphological progression of adjuvant induced arthritis in rats were further investigated by Hildebrandt et al. In their analysis, local irradiation of the arthritic joints reduced clinical symptoms, if given at days 15–19 after induction of arthritis by intradermal injection of mtb. Histopathological analysis performed at days 21 and 30 revealed a significant reduction of cartilage and bone destruction with minimal effect on the number of inflammatory cells in the periarticular tissue (Hildebrandt et al., 2000). In addition, the histologically observed prevention of disease progression appears to be related to a modulation of the iNOS activity with a reduction of the histochemical iNOS score and increase of heme oxygenase-1 (HO-1) expression (Hildebrandt et al., 2003).

Most of the analyses as reported before, however, used single fractions that exceed a dose of 1.0 Gy. In order to explore the lowest effective dose, the optimal time of treatment and the most favorable schedule, the effect of different fractionation schemes was analyzed by Liebmann et al. The most pronounced treatment effect was observed after two daily fractionated series of 5 × 0.5 Gy with an early treatment onset (days 10–14) and repetition after an interval of 8 days (days 22–26; Liebmann et al., 2004).

Relevantly, a variety of mechanisms, recognized in *in vitro* investigations to contribute to the immunomodulatory properties of LD-RT, were confirmed in the *in vivo* situation. Using an air pouch model in NMRI mice, the expression of TNF-α, IL-1β, and the proteins iNOS, HO-1, cyclooxygenase-2 (Cox2), and heat shock protein 70 (Hsp70) were investigated. Whereas the amount of exudates and number of inflammatory cells mainly remained unaffected, iNOS expression was decreased by irradiation concomitant with an increased expression of Hsp70 and HO-1 (Schaue et al., 2005). In addition, as binding of lymphocytes to blood vessel EC is crucial to drive an inflammatory process, the impact of LD-RT on adhesion and extravasation was analyzed by intra-vital microscopy in mice stimulated with LPS. In accordance to the *in vitro* findings, leukocyte adhesion in intestinal venules was diminished after irradiation with 0.1, 0.3, and 0.6 Gy, respectively (Arenas et al., 2006). This mechanistically correlated with increased levels of TGF-β_1_ in the serum of the mice, and partially could be restored by neutralization of the cytokine.

In a model of collagen inducible arthritis (CIA), DBA/1J mice were irradiated once before induction and on consecutive 4 weeks with a single dose of 0.5 Gy. The authors described a significant improvement of the clinical symptoms associated with a reduced amount of antitype II collagen antibodies and reduced values of inflammatory cytokines TNF-α, IFN-γ, and IL-6 in the serum of treated mice (Nakatsukasa et al., 2008). In proceeding analyses, the group further reported on a significant increased proportion of CD4(+)CD25(+)FoxP3(+) regulatory (Treg) cells in the spleen of irradiated mice at 4, 6, and 8 weeks after immunization with collagen and a hampered secretion of inflammatory IL-17 and IL-6 (Nakatsukasa et al., 2010). Tregs in turn are reported to act as suppressors of osteoclast cell activity, which drive arthritis and bone loss and therefore may contribute to the reduction of the clinical symptoms in mice treated with low-dose irradiation (Zaiss et al., 2010). In accordance to these observations, Weng et al. (2010) reported that a therapeutic effect of low-dose irradiation was associated with an increment in the proportion of Treg cells despite the overall reduction in lymphocyte count. By contrast, depletion of CD25 or folate receptor (FR)4(+) cells with specific antibodies before the treatment abolished the beneficial effects of irradiation confirming a fundamental role of Treg.

The models mentioned beforehand, however, suffer from the fact of an artificial induction of pathologic conditions in former healthy animals, that does in detail not reflect the situation in arthritic patients which are suffering from the disease since years and display an autoimmune status (Imboden, 2009). To overcome these limitations, Frey et al. (2009) were the first to analyze effects of low-dose irradiation in human TNF-α transgenic (hTNFtg) mice (Frey et al., 2009). These animals overexpress the cytokine TNF-α during their whole lifetime and develop a genetically determined polyarthritis (PA), with an onset after 3–6 weeks of age (beginning PA). The disease pattern in animals shows similarity to a human rheumatoid arthritis such as joint swelling and deformation, synovial inflammation, cartilage damage, and bone erosion, and is fully blown at an age of 9–12 weeks (Keffer et al., 1991). Irradiation of these mice with five times 0.5 Gy at a beginning (4–6 weeks) PA demonstrates significantly temporal improved clinical symptoms like a reduced paw swelling and increased grip strength. Those effects are less pronounced at later stages of the disease (9–12 weeks, fully established PA; Frey et al., 2009).

More insight on the impact of low-dose irradiation further arises from experiments on genetically determined MRL-lpr/lpr mouse autoimmune diseases. These animals are characterized by a deletion in the TNF receptor superfamily member six (FAS) gene resulting in an impaired apoptosis of autoreactive lymphocytes and aberrant T cell proliferation, concomitant with massive autoantibody production, immune complex glomerulonephritis, and arthritis. If irradiated with a daily single dose of 0.5 Gy for 4 weeks to a total dose of 10 Gy the mass of the spleen of the MRL-lpr/lpr mice was significantly reduced in line with a drastically reduced amount of CD3(+)CD4(−)CD8(−)B220(+) T cells, which drive the splenomegaly (Tago et al., 2008). In an additional model of experimental autoimmune encephalomyelitis (EAE) animals were irradiated with a total dose of 5.5 Gy, subdivided in 0.5 Gy fractions over 5 weeks (one fraction before induction and four fractions once a week for 4 weeks) resulting in a reduced EAE incidence along with a significant improved clinical score and delayed onset of pathological changes (Tsukimoto et al., 2008). These effects may arise from a hampered ability of spleenocytes to produce the pro-inflammatory cytokines IL-6, and IL-17. Furthermore, irradiated mice spleenocytes exhibited a reduced IFN-γ secretion and shifted the Th1/Th2 balance to an anti-inflammatory phenotype.

In summary, *in vivo* data and models clearly confirmed anti-inflammatory effects and have proven true a variety of immune modulatory effects of low-dose irradiation as summarized in Table 1. They may display suitable platforms for an intensified research of the underlying mechanisms and on options to improve the clinical efficacy of LD-RT.

**Table 1:** **Preclinical models and clinical/experimental parameters for the analyses of the anti-inflammatory effects of low-dose irradiation**.

Experimental model (**M**) Animal (**A**)	SD/TD	Time of irradiation	Clinical/biological effects	References
**M:** mechanically induced OA	SD 1.0 Gy	Different	↓ Inflammatory symptoms, ↓ pain ≈ degenerative changes	von Pannewitz (1933)
**A:** rabbit (knee joint)				
**M:** granugenol-induced OA	SD 1.5 Gy	1× Week immediately	↓ Joint swelling, ↓ cell proliferation within the SM, ↓ synovial fluid	Budras et al. (1986)
**A:** rabbit (knee joint)	TD 7.5 Gy	6, 12 Weeks p.i.		
**M:** zymosan/Mtb-induced OA	SD 1.0 Gy/SD 5.0 Gy	3 h p.i. or 4× daily	Zymosan: ↓ joint swelling ↓↓ cartilage/bone destruction Mtb: ↓↓ joint swelling (4× 1 Gy), ↑↑ bone destruction (1× 5 Gy)	Trott et al. (1995)
**A:** Wistar rat	TD 4.0 Gy/TD 5.0 Gy			
**M:** papain-induced OA	SD 1.0 Gy	24 h p.i.	↓↓ Joint diameter, ↓ SM-thickness and cell layers, ↓ distance between vessels and SM	Fischer et al. (1998)
**A:** rabbit (knee joint)	TD 5.0 Gy	5× Daily		
**M:** Mtb-induced RA	SD 1.0 Gy/TD 5 Gy	Day 15 p.i.	↓↓ HPV, ↓↓ AS, ↓ ESR ↓↓ cartilage- and bone destruction, ↓ iNOS expression, ≈inflammatory infiltration	Hildebrandt et al. (2000, 2003)
**A:** Lewis rat	SD 0.5 Gy/TD 2.5 Gy	5× daily		
**M:** Mtb-induced RA	SD 0.5 Gy/SD 1.0 Gy	Days 10, 15, or 22 p.i	↓ HPV, ↓ AS, early IR (acute, day 10), and 0.5 Gy most effective. IR (chronic days 22–26) ≈ clinical signs	Liebmann et al. (2004)
**A:** Lewis rat	TD 2.5/TD 5.0 Gy	1× SD/ 5 days (FS1)		
		5× SD/9 days (FS2)		
**M:** carrageenan air pouch model	SD 0.5/1.0/2.0/5.0 Gy	6 h after challenge	≈Inflammatory exudates and cell number ↓ iNOS expression, ↓ IL-1β ↑ Hsp70, ↑ HO-1	Schaue et al. (2005)
**A:** NMRI mice				
**M:** LPS induced systemic response	SD 0.1/0.3/0.6 Gy	1 h before LPS challenge	↓ Leucocyte adhesion in intestinal venules (max. 0.3 Gy), ↑ circulating levels of TGF-β1	Arenas et al. (2006)
**A:** C57/Bl/6 mice				
**M:** systemic lupus erythematosus	SD 0.5 Gy	24 h before induction and 4× week	↓ Weight of spleen, ↓ CD(+)CD4(+)CD8(−) B220(+) Tcells, ↑ FoxP3 T(reg) in spleen, ↓ anti-DNA antibodies	Tago et al. (2008)
**A:** MRL-lpr/lpr mice	TD 2.5 Gy			
**M:** collagen induced RA	SD 0.5 Gy	24 h before RA induction and 4× week	↓ Clinical symptoms, ↓ joint collapse, cytokines TNF-α, IFN-γ, IL-6 in serum	Nakatsukasa et al. (2008)
**A:** DBA/1 mice	TD 2.5 Gy			
**M:** autoimmune encephalomyelitis (EAE)	SD 0.5 Gy	24 h before EAE induction and 4× week	↓EAE incidence, ↓clinical score, ↓ TNF-a, IL-6, IL-17 in spleen, ↑ FoxP3 T(reg) in spleen, ↓ IFN-γ in serum	Tsukimoto et al. (2008)
**A:** SJL/J mice	TD 2.5 Gy			
**M:** genetically determined RA	SD 0.5 Gy	6–7 weeks and 10–12 weeks	↓↓ Ankle swelling, ↑grip strength at the beginning PA	Frey et al. (2009)
**A:** (hTNFtg) mice	TD 2.5 Gy			
**M:** collagen induced RA	SD 0.5 Gy	24 h before RA induction and 4× week	↓ Arthritis score, ↓ antitype II collagen ab, ↑ FoxP3(+) T(reg) in spleen, ↓ IL-6, IL-17	Nakatsukasa et al. (2010)
**A:** DBA/1 mice	TD 2.5 Gy			
**M:** collagen induced RA	SD 0.4 Gy	24 h before or at day 25	↓ AS, ↓ number of lymphocytes in circulation, ↑ FoxP3 T(reg) in circulation	Weng et al. (2010)
**A:** DBA/1 mice				

*A, animal; AS, arthritis score; EAE, experimental autoimmune encephalomyelitis; ESR, erythrocyte sedimentation rate; HO-1, heme oxygenase; HPV, hind paw volume; hTNFtg, human TNF transgenic mice; Hsp70, heat shock protein70; IL-1, Interleukin-1; iNOS, inducible nitric oxide synthase; LPS, lipopolysaccharide; M, experimental model; Mtb, mycobacterium tuberculosis; IR, ionizing radiation; OA, osteoarthritis; RA, rheumatoid arthritis; SD, single dose; SM, synovial membrane; TD, total dose; TGF-β_1_, transforming growth factor β1; ↑, increase; ↓, decline; ≈, not changed; ↑↑ highly significant increase; ↓↓ highly significant decline*.

## Conclusion

**Figure 1:**
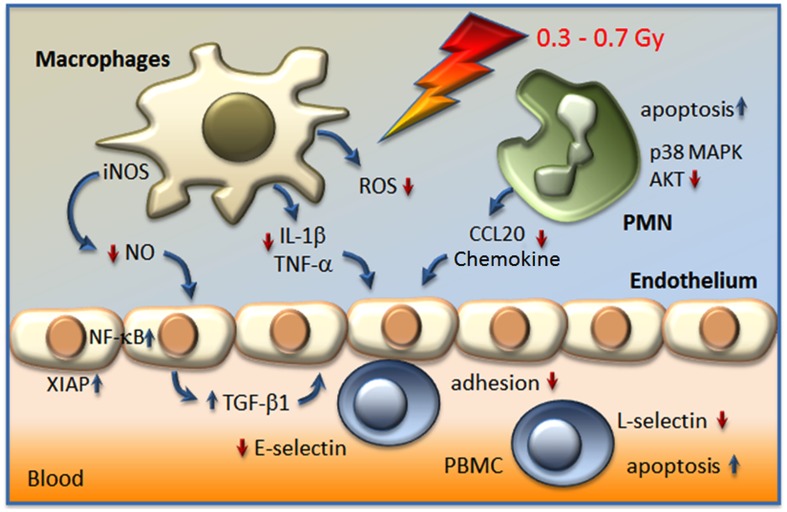
**Actual model on the modulation of inflammatory cell activity and factors involved in the anti-inflammatory effect of LD-RT (<1 Gy)**. Irradiation resulted in a hampered adhesion of peripheral blood mononuclear cells (PBMC) to the endothelium, due to the secretion of the anti-inflammatory cytokine transforming growth factor β1 (TGF-β1), a decreased expression of E-selectin on the surfaces of endothelial cells, a local increase of apoptosis, and the proteolytic shedding of L-selectin from PBMC. In stimulated macrophages a diminished activity of the inducible nitric oxide synthase (iNOS) in line with reduced levels of nitric oxide (NO), a lowered production of reactive oxygen species (ROS), and a diminished secretion of interleukin-1β (IL-1β) and tumor necrosis factor-α (TNF-α) may contribute to local anti-inflammatory effects. Moreover, polymorphonuclear cells (PMN) respond to low-dose exposure with a locally increased rate of apoptosis, a hampered secretion of CCL20 chemokine and alterations in signal transduction pathways p38 mitogen activated protein kinase (MAPK) and protein kinase B (AKT).

Considerable progress has recently been achieved in the understanding of cellular targets and molecular mechanisms involved in the immune modulatory properties of low-dose irradiation as depicted in Figure 1. However, as (chronic) inflammatory and degenerative diseases are based upon complex (patho)physiological networks, a variety of yet unresolved questions exists. Thus, intensive translational and clinical research efforts as well as the development of further basic models are seriously needed to recognize additional contributing factors and mechanisms. Moreover, on-going efforts should also focus on a putative relationship to tumor immune biology to optimize clinical use of radiation therapy of both benign and malignant diseases.

## Conflict of Interest Statement

The authors declare that the research was conducted in the absence of any commercial or financial relationships that could be construed as a potential conflict of interest.
